# In this month’s Bulletin

**DOI:** 10.2471/BLT.17.001017

**Published:** 2017-10-01

In the editorial section, Christopher Dye and Shambhu Acharya (666) announce a theme issue on health in the sustainable development goals. Alarcos Cieza et al. (667) announce an upcoming theme issue on programmes to detect, prevent and treat visual impairments from all causes.

In the news section, Tatum Anderson (670–671) reports on efforts to train public health professionals in effective responses to false information about vaccines. Tabaré Vázquez (672–673), president of Uruguay, describes health reforms taken to reduce noncommunicable diseases.

## Australia

### Reducing brand-recognition 

Janet Hoek et al. (726–728) encourage more countries to insist on plain packaging for cigarettes.

## Chile

### Myocardial infarcts and smoking legislation 

Carolina Nazzal and Jeffrey E. Harris (674–682) track parallel trends in smoking bans and cardiovascular outcomes.

## India

### Adjusting to motherhood

Ravi Prakash Upadhyay et al. (706–717) establish prevalence estimates for postpartum depression. 

## Kenya

### Providing health care to mothers and children 

Isabella Maina et al. (683–694) use health facility data to assess subnational coverage of services.

## Nepal

### Surviving snakebite

Bhola R Shrestha et al. (718–719) argue for an effective antivenom.

## Papua New Guinea

### Vanishing malaria

Manuel W Hetzel et al. (695–705) track a decrease in malaria prevalence from 2008 to 2014.

## Samoa

### Health costs of cheap calories

Anne Marie Thow et al. (723–725) describe an import ban on turkey tails.

## Global

### Improved drug price negotiations

Alessandra Ferrario et al. (720–722) describe national collaboration on medicines procurement.

